# Tunicamycin Sensitivity-Suppression by High Gene Dosage Reveals New Functions of the Yeast Hog1 MAP Kinase

**DOI:** 10.3390/cells8070710

**Published:** 2019-07-12

**Authors:** Mariana Hernández-Elvira, Ricardo Martínez-Gómez, Eunice Domínguez-Martin, Akram Méndez, Laura Kawasaki, Laura Ongay-Larios, Roberto Coria

**Affiliations:** 1 Departamento de Genética Molecular, Instituto de Fisiología Celular, Universidad Nacional Autónoma de México, CP 04510 Cd., Mexico; 2 Unidad de Biología Molecular, Instituto de Fisiología Celular, Universidad Nacional Autónoma de México, CP 04510 Cd., Mexico

**Keywords:** yeast, Hog1, MAP kinase, endoplasmic reticulum, stress, suppression, unfolded protein response (UPR)

## Abstract

In the yeast *Saccharomyces cerevisiae*, components of the High Osmolarity Glycerol (HOG) pathway are important for the response to diverse stresses including response to endoplasmic reticulum stress (ER stress), which is produced by the accumulation of unfolded proteins in the lumen of this organelle. Accumulation of unfolded proteins may be due to the inhibition of protein *N*-glycosylation, which can be achieved by treatment with the antibiotic tunicamycin (Tn). In this work we were interested in finding proteins involved in the ER stress response regulated by Hog1, the mitogen activated protein kinase (MAPK) of the HOG pathway. A high gene dosage suppression screening allowed us to identify genes that suppressed the sensitivity to Tn shown by a *hog1Δ* mutant. The suppressors participate in a limited number of cellular processes, including lipid/carbohydrate biosynthesis and protein glycosylation, vesicle-mediated transport and exocytosis, cell wall organization and biogenesis, and cell detoxification processes. The finding of suppressors Rer2 and Srt1, which participate in the dolichol biosynthesis pathway revealed that the *hog1Δ* strain has a defective polyprenol metabolism. This work uncovers new genetic and functional interactors of Hog1 and contributes to a better understanding of the participation of this MAPK in the ER stress response.

## 1. Introduction

The synthesis of transmembrane and secreted proteins occurs in ribosomes attached to the endoplasmic reticulum (ER) membrane. In the ER lumen such proteins are subjected to different modifications such as *N*-glycosylation, formation of disulphur bonds and protein folding. Properly processed proteins continue through the secretion pathway in order to be transported to their final location. All organisms are exposed to adverse conditions that decrease the capacity of the ER to fulfill these processes, leading to the production and accumulation of misfolded proteins, inducing a condition known as endoplasmic reticulum stress (ER stress). ER stress triggers a cellular response that includes the unfolded protein response (UPR), which is a conserved signaling pathway present in all eukaryotic cells, including yeast [1], *Dictyostelium discoideum* [2], plants [3], and mammals [4]. In the yeast *Saccharomyces cerevisiae* the UPR consists of an endoplasmic reticulum membrane sensor named Ire1 [5], which in the presence of misfolded proteins oligomerises and autophosphorylates [6,7]; this in turn triggers its cytoplasmic endonuclease activity, which processes the *HAC1* pre-mRNA [8]. The spliced mRNA is efficiently translated to produce the transcription factor Hac1 [8,9], which regulates transcription of several target genes, such as those encoding chaperones, glycosylation enzymes and proteases among others [10] in order to alleviate the accumulation of unfolded proteins.

It has been proposed that the UPR is not the only cellular pathway needed to cope with the ER stress [11]. Components of other pathways appear to be required to produce a full cellular response to ER stress inducers; particularly, some components of the high osmolarity glycerol (HOG) pathway have been implicated in the response to ER stress induced by the antibiotic tunicamycin (Tn). This antibiotic is a nucleoside structurally similar to the UDP-*N*-acetylglucosamine and blocks the first step of the *N*-glycosylation catalyzed by the Glucosamine-*N*-acetil phosphotransferase enzyme [12,13]. The HOG pathway is a signaling system involved in survival under hyperosmotic conditions and it consists of two mechanistically different branches connected to a MAPK module, the SLN1 branch and the SHO1 branch, which converge on the scaffold MAPKK Pbs2, which in turn activates the MAPK Hog1 [14,15,16]. Once activated, Hog1 is translocated into the nucleus [17,18] where it regulates expression of genes whose products are involved in cell cycle arrest and adaptation to high osmolarity conditions.

In order to respond to ER stress induced by Tn, the cell requires the presence of both Pbs2 and Hog1. Additionally, Ssk1, the phosphorelay response regulator of the SLN1 branch, appears to be required for Tn response; however, components of the SHO1 branch seem to be dispensable [12,13]. As mentioned above, the presence of Hog1 is required for a proper response to Tn, but it appears that it does not need to be phosphorylated in order to produce resistance to the ER stress inducer. Indeed, a strain expressing an unphosphorylated form of Hog1 is able to grow in Tn, although not at the wild type level, but clearly above the growth shown by the *HOG1* null mutant [13]. Additionally, treatment with high Tn concentrations for an extended period of time only produces a very weak level of Hog1 phosphorylation that does not trigger its nuclear import [13]. Interestingly, a full cellular response to Tn requires the kinase activity of Hog1, since a point mutation that eliminates its kinase activity produces high sensitivity to Tn [13]. In addition to its participation in the ER stress response, several reports suggest that Hog1 has cytoplasmic activities, for example in the general stress response system [19,20] and in regulation of mitophagy [21,22].

With the aim of increasing knowledge regarding the participation of Hog1 in the ER stress response, we searched for genes whose products can suppress the sensitivity to Tn displayed by a Hog1-deficient strain but not its sensitivity to hyperosmotic stress. With this screening, we not only detected genes that were able to specifically suppress the Tn sensitivity of the *hog1Δ* mutant, but we also found genes that reverted the sensitivity shown by the *hog1Δ* mutant and by the *hac1Δ* mutant.

## 2. Materials and Methods

### 2.1. Yeast Strains, Gene Disruptions, and Culture Conditions

The *S. cerevisiae* strains used in this work are shown in Supplementary Table S1. All of them are isogenic to BY4742 or BY4741. The wild type and the single null mutants were obtained from the EUROSCARF collection. Double mutants were constructed by integration of the *NATMX4* cassette (which provides nourseothricin resistance) into the *HOG1* locus of single mutants. The integrating cassette was constructed by PCR using the oligonucleotides DHOG1F and DHOG1R (Supplementary Table S2) and contained the *NATMX4* gene flanked by *HOG1* 5′ and 3′ UTR fragments of 40 base pairs respectively. Integrations of the cassette were performed by homologous recombination. All deletions were confirmed by PCR.

Yeast cells were grown at 30 °C in YPD medium (1% yeast extract, 2% peptone, and 2% glucose) or SD medium (0.67% yeast nitrogen base without amino acids and 2% glucose, supplemented with the required amino acids for plasmid selection). Glucose was substituted by 2% raffinose (Sraf) or 2% galactose (Sgal) according to the requirements of the experiments. When needed, nourseothricin (100 µg/mL) was added to the media. YPGAL was the same as YPD except for the substitution of glucose by galactose.

*Escherichia coli* DH5α strain was used to propagate plasmids. Bacteria were grown at 37 °C in LB medium (1% tryptone, 0.5% yeast extract, and 0.5% NaCl) containing 100 µg/mL ampicillin or 50 µg/mL kanamycin (for library-plasmid selection).

### 2.2. Stress Assays

Stress sensitivity assays were done by dropping aliquots of 10-fold serial dilutions of early-logarithmic YPD-cell cultures (OD_600_ adjusted to 0.05) on Tn and KCl plates. Plates were incubated at 30 °C for 72 h and scanned. For liquid cultures, cells with the same density were inoculated in triplicate in Honeycomb 2 plates (100 wells). Density readings were recorded every hour during 48 h of incubation at 30 °C in a BioScreen C microplate spectrophotometer (Oy Growth Curves Ab Ltd, Helsinki, Finland). The relative growth rate is the slope value of the linear regression of three independent liquid cultures.

### 2.3. High Dosage Suppression Screening

**Figure 1:**
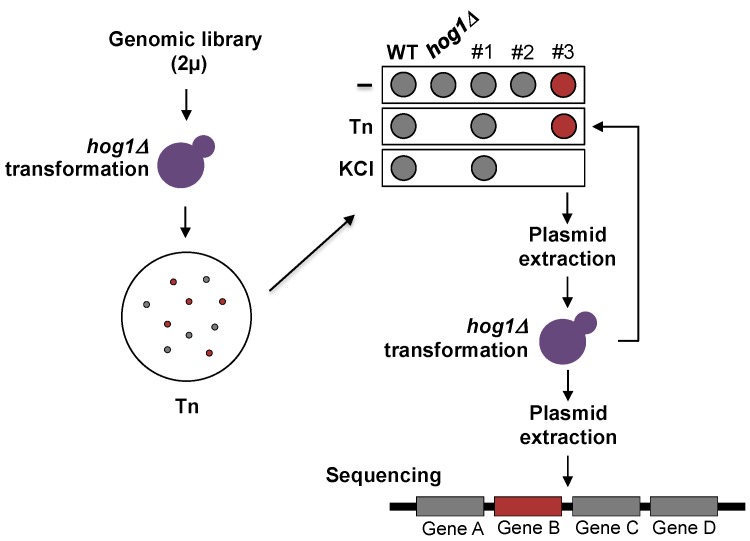
Dosage suppression screening protocol. The *hog1∆* strain was transformed with a genomic library constructed in a 2 µ plasmid. Yeast cells were plated on selective medium (SD + 25 µg/mL of the required amino acids and nitrogen bases) containing 1 µg/mL Tn. Plates were incubated for 48 h at 30 °C. Colonies that appeared were grown to early-logarithmic phase in liquid selective medium and adjusted to 0.5 OD_600_. An aliquot of each transformant was dropped onto selective medium containing either 0.5 µg/mL Tn or 1M KCl. Plasmids were purified from the Tn-resistant and KCl-sensitive clones and reintroduced into the *hog1Δ* strain for a second round of selection. Suppressor plasmids were extracted, and the genomic inserts were sequenced.

A diagram representing the suppression-screening assay is shown in Figure 1. The *hog1∆* mutant was transformed with a genomic library constructed to specifically perform overexpression screenings in *S. cerevisiae* [23]. The library contains a collection of more than 1000 overlapping genome fragments from the FY4 *S. cerevisiae* strain, cloned into the multicopy pGP564 plasmid. Transformants were plated in selective medium containing 1 µg/mL Tn. Tn resistant clones were streaked on 1 M KCl and those sensitive to high osmolarity were selected. Plasmids from Tn resistant/KCl sensitive cells were extracted and propagated in *E. coli*. The selected plasmids were re-introduced into *hog1Δ* yeast cells for a second round of selection. Ends of the yeast genome inserts were sequenced in order to map the Tn suppressor genome fragments. Standard yeast genetics protocols were used [24].

### 2.4. Gene Expression

Each ORF contained in the suppression plasmids was cloned with its respective promoter in order to be expressed in yeast cells. Subcloning was done by PCR using oligonucleotides shown in Supplementary Table S2 (only the oligonucleotides for suppressor genes are shown). The PCR products were obtained using genomic DNA of the BY4742 wild type strain as template. The PCR products were cloned into the pGEM-T-Easy vector and then subcloned into the yeast YEp352 vector [25]. The YEp352 clones were used for yeast transformation. All PCR products were sequenced in full.

### 2.5. Physical Interactions and Construction of the Interaction Network

Physical protein interactions between Hog1 and suppressor proteins were determined by the Bimolecular Fluorescence Complementation (BiFC) assay [26]. Proteins fused to the amino-end (VN) of the fluorescent protein Venus were obtained from the VN-fusion library (Bioneer, Daejeon, Republic of Corea) that is constructed in the BY4741 (*Mata*) strain. Hog1-VC (carboxyl-end of the Venus protein) was constructed by fusing the *HOG1* ORF in frame with the gene encoding VC. The fused gene was integrated into the *HOG1* locus of the BY4742 (*Matα*) strain by homologous recombination. Co-expression of VN and VC fusion constructs was achieved in diploid cells after mating BY4741 and BY4742 recombinant strains. Protein interactions were detected by flow cytometry using the Attune Acoustic Focusing Cytometer. Fluorescence statistics were calculated with the Attune software (version 2.1) and the statistical significance was determined with a Welch *t*-test.

Data of the known interactors for each suppressor gene and *HOG1* were obtained from the BioGRID database (https://thebiogrid.org/) (version 3.4.144), which is a repository of available physical and genetic interactions determined by different low- and high-throughput experimental assays and compiled through comprehensive curation [27]. The physical interaction data were extracted and visualized with Cytoscape [28] according to the protocol previously described [29]. The output interactors, i.e., the proteins that interact with only one node of the network, were eliminated in order to select the proteins connected with at least two other interactors. The network obtained after this reduction was clustered with the force directed layout with default parameters in Cytoscape and the visualization (separation of the nodes) was improved manually. The edges were colored according to the experimental assay that supports each interaction in the database.

### 2.6. Polyprenol Determination

Yeast cells were grown to mid-logarithmic phase in YPGAL (OD_600_ adjusted to 0.5) and incubated during 2 h with either 1µg/mL Tn or DMSO (Tn dissolvent). Yeast cells were centrifuged and the pellet was washed with water. Polyprenols extraction was performed as previously described [30] with modifications. Yeast cells were resuspended in water and disrupted with glass beads. The lipids were extracted by shaking during 2 h with chloroform/methanol (2:1 *v*/*v*) at RT. The organic phase was collected washed three times with 1/5 volume of 10mM EDTA in 0.9% NaCl and evaporated to dryness. Lipids were resuspended in methanol/water (10:1 *v*/*v*) containing 15% KOH and alkaline hydrolysis was performed during 2 h at 95 °C. Lipophylic products were extracted twice with diethyl ether and evaporated to dryness. The lipids obtained were dissolved in hexane and applied to a silica column equilibrated with hexane. The column was washed with 3% diethyl ether in hexane and the polyprenol fraction was eluted with 17% diethyl ether in hexane. The fraction was evaporated to dryness, resuspended in hexane and subjected to HPLC analysis. Polyprenols detection was performed with a reverse phase Symmetry C18 Waters column (3.5 µm, 4.6 × 75 mm) using a HPLC Waters Alliance e2695/2489/2414 equipment (Milford, MA, USA). Samples were run at 25 °C at a flow rate of 1.2 mL/min in isocratic elution with 55% methanol, 23% isopropanol, and 22% hexane and detected with UV light (210 nm). We used 1 μg/μL of C-90 Dolichol (Dolichol-18) from Larodan Research Grade Lipids (Solna, Sweden) as a standard. Polyprenol concentration corresponded to the area under the curve of each peak, referred to the area under the curve of the internal standard peak and expressed by mg of protein. Mean values, standard deviation, and significance were graphed and analyzed from three independent experiments. Statistical significance was tested with Student *t* test. 

## 3. Results

### 3.1. Isolation of High Dosage-Gene Suppressors of Hog1Δ Tunicamycin Sensitivity

In order to shed light onto the way in which Hog1 participates in the response to ER stress inducers, we designed a dosage-suppression screening to identify genes required for the Hog1-Tn response. For the screening the *hog1Δ* mutant was transformed with a genome library constructed in a high copy plasmid [23] selecting clones resistant to Tn but sensitive to KCl. To this end, yeast transformants were plated in medium containing Tn to isolate cells resistant to the antibiotic; then, the transformants were dropped on plates containing KCl in order to identify the clones that were sensitive to hyperosmotic stress (Figure 1). The suppressor plasmids were extracted from the selected transformants and were reintroduced into the *hog1Δ* mutant to confirm Tn resistance and KCl sensitivity (Figure 1).

**Figure 2:**
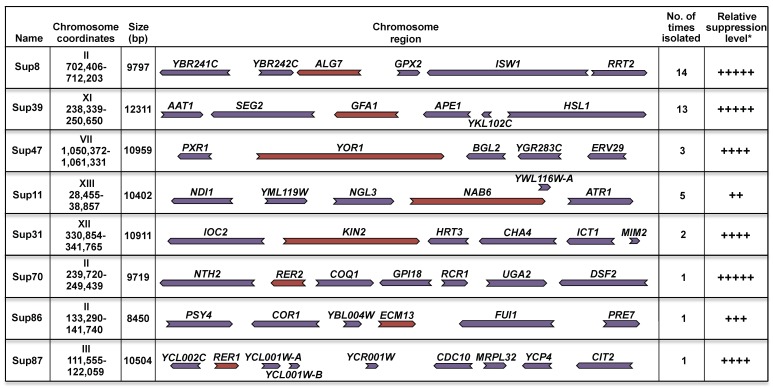

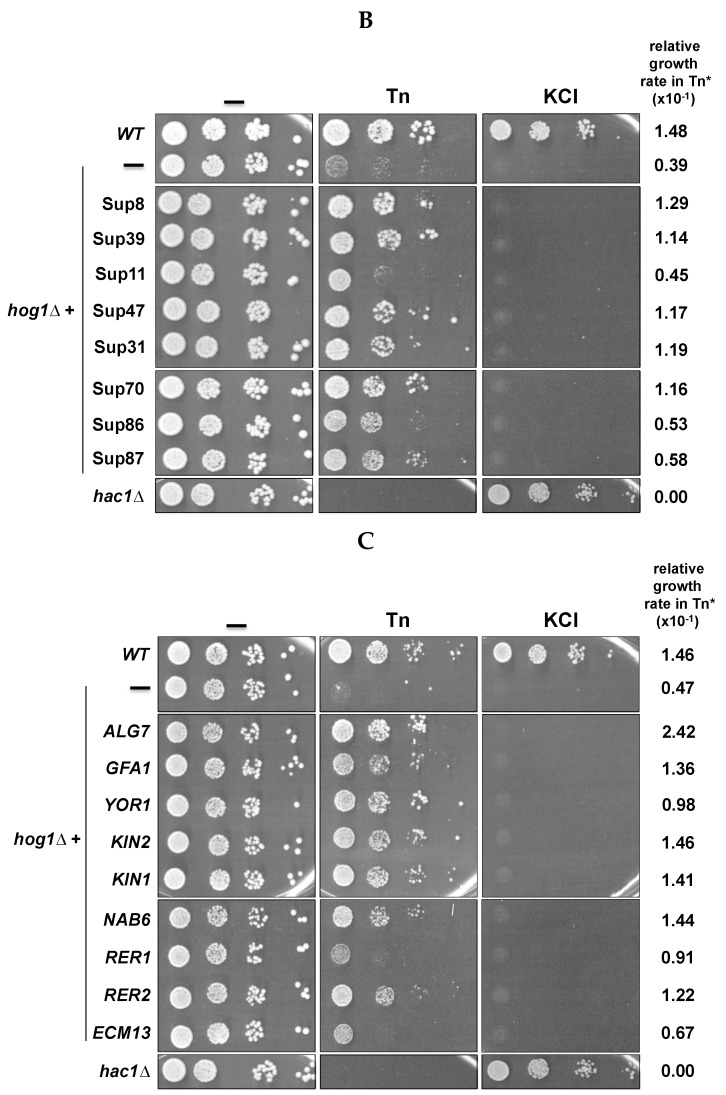
Suppression of the *hog1∆* Tn sensitivity. (**A**) Schematic representation of the genomic region contained in each suppressor plasmid. Chromosome number and insert coordinates and size are shown. Number of isolated times that each suppressor plasmid was isolated along with its relative suppression level (according to Figure 2B) are indicated. ORF orientation is depicted by the colored arrows. ORFs that show suppressor activity are marked in brown. (**B**) Serial dilution dropping assay of the *hog1∆* strain transformed with suppressor plasmids. Yeast cells were grown in YPD to early-logarithmic phase and adjusted to 0.5 OD_600_ in fresh YPD. 10-fold serial dilutions were spotted on selective medium (see legend of Figure 1) containing either 0.5 µg/mL Tn or 1 M KCl. The *hac1∆* mutant was used as Tn sensitivity control. Plates were incubated at 30 °C for 72 h and scanned. (**C**) Genes colored in brown in Figure 2A were subcloned into the multicopy plasmid YEp352 and expressed in the *hog1∆* strain under the control of their own promoter. A serial dilution assay was performed as described in Figure legend 2B. (*) The relative growth rate is the slope value of the linear regression of liquid cultures made in triplicate and grown in YPD containing 0.5 µg/mL Tn. Liquid cultures were performed in a microplate spectrophotometer at 30 °C with constant shaking. The OD_600_ readings were determined at 1 h intervals during 48 h. *KIN1*, which was not detected in the screening was included in this assay since it is paralogous to *KIN2* (see text).

With this strategy, we isolated 40 plasmids that conferred resistance to Tn but not to hyperosmotic stress. These plasmids were sequenced to determine the chromosomal region responsible for the suppression. Among these 40 plasmids only 8 different regions were represented (Figure 2A). The regions are distributed in 6 different chromosomes and their size range is from 9 Kb to 12 Kb. Figure 2A summarizes the characteristics of the 40 suppressor plasmids; suppressor 8 was the most represented (14 times), while suppressors 70, 86, and 87 were isolated only one time. The level of Tn sensitivity suppression varied between strains. Sup 11 showed the weakest suppressor level while suppressors 8, 39, and 70 showed the strongest (Figure 2A,B). In our assays we included as a control the *hac1Δ* mutant, which lacks the basic leucine zipper (b-Zip) transcription factor, which regulates the gene transcription in the UPR pathway. This mutant is highly sensitive to Tn (Figure 2B).

Since each chromosomal region contained several ORFs (Figure 2A), we subcloned each of them independently in order to identify the ORF responsible for the suppression. From this assay we identified 8 genes (colored in brown in Figure 2A) that conferred resistance to Tn but not to hyperosmotic stress upon the *hog1Δ* mutant (Figure 2C). The level of suppression varied among the genes. We found differences in sensitivity to Tn and therefore in the growth rate of the *hog1Δ* mutant transformed with the different genes (Figure 2C). The highest growth rate of the *hog1Δ* mutant in Tn was observed with *ALG7*, while the lowest was obtained with *ECM13*. Although *KIN1* was not found as a suppressor in this screening, it was included in this study since it has been reported to be paralogous to *KIN2* [31]. As can be observed, *KIN1* and *KIN2* had the same level of suppression (Figure 2C).

### 3.2. Genes Can Be Divided into General and HOG-Specific Suppressors of Tn Sensitivity

**Figure 3:**
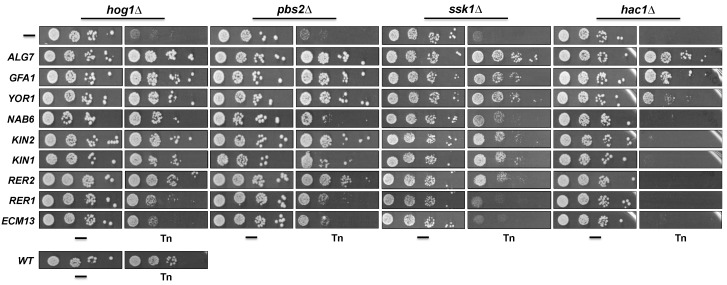
Effect of expression of suppressor genes in the Tn sensitivity of different strains. The suppressor genes were overexpressed in the indicated strains as described in Figure legend 2C. A serial dilution assay was performed as described (Figure legend 2B). Plates were incubated at 30 °C for 72 h and scanned.

In order to determine whether the isolated genes were specific suppressors of the Tn-sensitivity observed in the *hog1Δ* mutant, we tested their suppressor activity in mutants of other components of the HOG pathway that have been implicated in the response to ER stress induced by Tn, specifically *PBS2* and *SSK1* [13]. We also introduced the suppressor genes in the *hac1Δ* in order to ascertain whether suppression is or is not restricted to the components of the HOG pathway. We observed that all suppressor genes did confer resistance, to different degrees, upon the *pbs2Δ* and *ssk1Δ* mutants. Interestingly, three genes, *ALG7*, *GFA1* and *YOR1*, also conferred resistance upon the *hac1Δ* mutant (Figure 3). This indicates that *ALG7*, *GFA1* and *YOR1* are general suppressors of Tn sensitivity, while *NAB6*, *KIN1*, *KIN2*, *RER1*, *RER2* and *ECM13* can be considered specific suppressors of the deficiency shown by the mutants of the HOG pathway. The general suppressors were the most represented in the isolated plasmids (30 of 40) (Figure 2B). In contrast, the specific suppressors were represented in only 10 of 40 isolated plasmids (excluding *KIN1*), suggesting that these suppressors can be found more rarely in this kind of screening. Table 1 summarizes the main characteristics of the general and specific suppressors including the proved or putative cellular process where they can be participating (Saccharomyces Genome Database (SGD): www.yeastgenome.org; Gene Ontology (GO) database: www.geneontology.org). Regarding the general suppressors, *ALG7* encodes the UDP-*N*-acetyl-glucosamine-1-P-transferase which catalyzes the first step of *N*-glycosylation and is a direct target of Tn [32,33]; *GFA1* encodes the glutamine-fructose-6-phosphate amidotransferase which participates in the synthesis of chitin, a constituent of the yeast cell wall, and in the synthesis of *N*-acetylglucosamine [34,35]; and *YOR1* codes for an ATP-binding cassette (ABC) transporter located in the plasma membrane [36,37] which participates in cell detoxification processes. Regarding the specific *hog1Δ* suppressors, these include genes that code for proteins with a variety of functions. *NAB6* encodes a protein that binds polyadenylated RNAs which mainly encode proteins destined to the cell wall [38]; *KIN2* and its paralogous *KIN1* encode serine/threonine protein kinases that have been implicated in exocytosis and may also play a role in septin cytoskeleton and cell wall organization [39,40]; *RER2* encodes the *cis*-prenyltransferase involved in the production of dolichol from the isoprenoid lipid farnesyl diphosphate [41]. Dolichol is the acceptor and carrier of oligosaccharides that are transferred to proteins during the folding process. *RER1* encodes a protein known as Retention in the Endoplasmic Reticulum which participates in protein retention in the ER lumen and in retrograde vesicle-mediated transport from Golgi to ER [42,43]; and *ECM13* encodes a protein of unknown function that is upregulated by cell wall damage and appears to be involved in cell wall biosynthesis and architecture [44,45].

### 3.3. Inactivation of Some Suppressor Genes Confers Sensitivity to Tn

**Figure 4:**
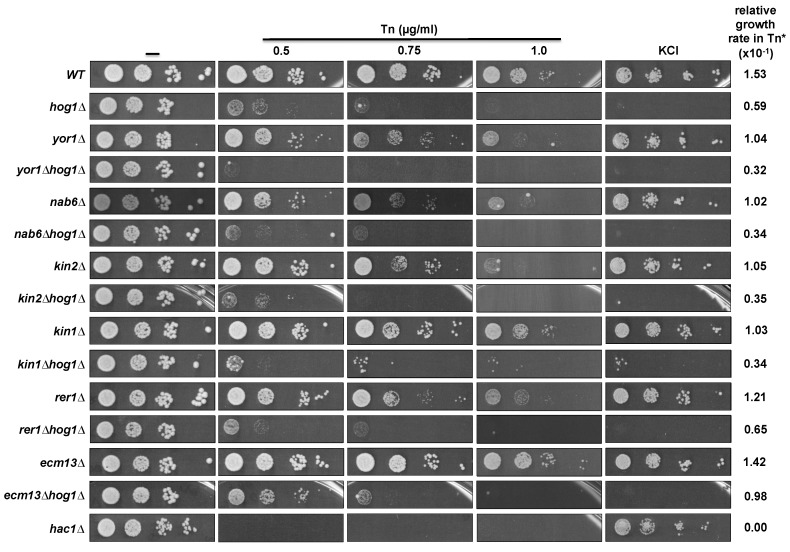
Effect of the inactivation of the suppressor genes on Tn sensitivity and their epistatic interaction with *HOG1*. Single mutants of each suppressor ORF (obtained from the EUROSCARF collection) and the double mutants (constructed as described in the methods section), were tested in different concentrations of Tn or 1M KCl. Yeast cells were grown in YPD and plated as described in Figure legend 2B. Plates were incubated at 30 °C for 72 h and scanned. (*) Relative growth rate was determined from growth at 0.5 µg/mL Tn as described in Figure legend 2.

We next evaluated whether the inactivation of some of the suppressor genes affects cell growth in Tn and KCl. In this assay we did not include *ALG7*, *GFA1*, and *RER2* null mutants since *ALG7* and *GFA1* are essential genes and the *rer2Δ* mutant has a severe growth impairment. It was evident that disruption of *YOR1*, *NAB6*, and *KIN2* induced sensitivity to Tn at the highest concentration tested (1 µg/mL), while sensitivity was barely detected at lower concentrations (Figure 4). Disruption of *RER1* also induced sensitivity to Tn but to a lesser extent compared to disruption of *YOR1, NAB6*, and *KIN2*, while inactivation of the *ECM13* gene did not affect growth in Tn. In contrast to the *kin2Δ* mutant, the *kin1Δ* mutant showed very low sensitivity to Tn. As expected, none of the null mutants of the suppressor genes showed growth defects when they were plated on 1M KCl (Figure 4). Additionally, we detected that inactivation of *YOR1*, *NAB*6, *KIN2*, and *KIN1* in a *hog1Δ* background increased the sensitivity to Tn shown by the *hog1Δ* mutant (Figure 4), suggesting that they might participate in Hog1 independent pathways during the Tn response. In contrast, inactivation of *RER1* and *ECM13* did not affect the Tn sensitivity of the *hog1Δ* mutant, suggesting that they may be components of the same pathway.

### 3.4. Suppressor Genes may Form an Interaction Network with HOG1

The genes that suppress the Tn sensitivity of the *hog1Δ* strain do not appear to encode canonical interactors of Hog1. However, it could be that a putative interaction with one or more suppressor proteins would occur in conditions that induce ER stress. Therefore, we looked for physical interactions between Hog1 and the specific suppressor proteins in the presence of Tn. Using Hog1 as bait and the specific suppressor proteins Nab6, Kin1, Kin2, Rer1, Rer2, and Ecm13 as prays we performed a Bimolecular Fluorescence Complementation (BiFC) assay to detect physical interactions. As expected, none of the suppressor proteins tested appeared to interact physically with Hog1 with and without Tn exposure (Supplementary Table S3), although the fluorescence displayed by the Kin2-Hog1 and Rer2-Hog1 pairs under un-induced conditions was slightly higher than the negative control (Supplementary Table S3). This observation suggests that the suppressors could require one or more intermediates to associate physically with Hog1. 

**Figure 5:**
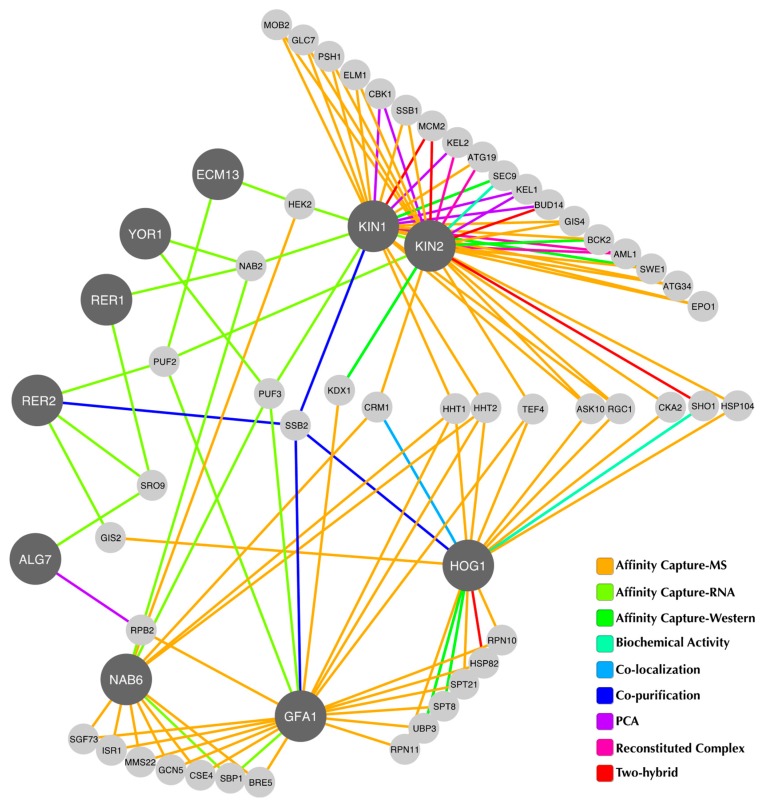
Physical interaction network. Physical interactions of suppressor proteins and Hog1 as deduced from the BioGRID database and visualised with Cytoscape. The network was constructed according to the criteria described in the methods section. Hog1 and suppressor proteins are depicted with dark grey circles. The connecting lines represent the physical interactions and are colored according to the experimental assay that supports each interaction. Known interactors found in the database are colored in light grey.

In order to determine whether or not the suppressor genes could participate in functional processes required to cope with the ER stress induced by Tn, we constructed a physical interaction network based on available data from the BioGRID database (Figure 5). This analysis showed that five suppressors, namely Kin1, Kin2, Rer2, Gfa1, and Nab6 may indeed interact with Hog1 through one intermediate. For Kin1, Rer2, and Gfa1, the intermediate protein is Ssb2, a cytoplasmic chaperon of the Hsp70 family that is involved in protein folding processes [46]. Interestingly, Rer2 may also have a physical interaction with Hog1 through Gis2, a RNA binding protein present in processing bodies and stress granules, which contain translationally repressed RNAs [47]. Although the suppression mechanism cannot be deducted from the structure of the interaction network, it provides relevant information regarding proteins that belong to pathways linked with the ER stress response and not previously associated with Hog1. 

**Figure 6:**
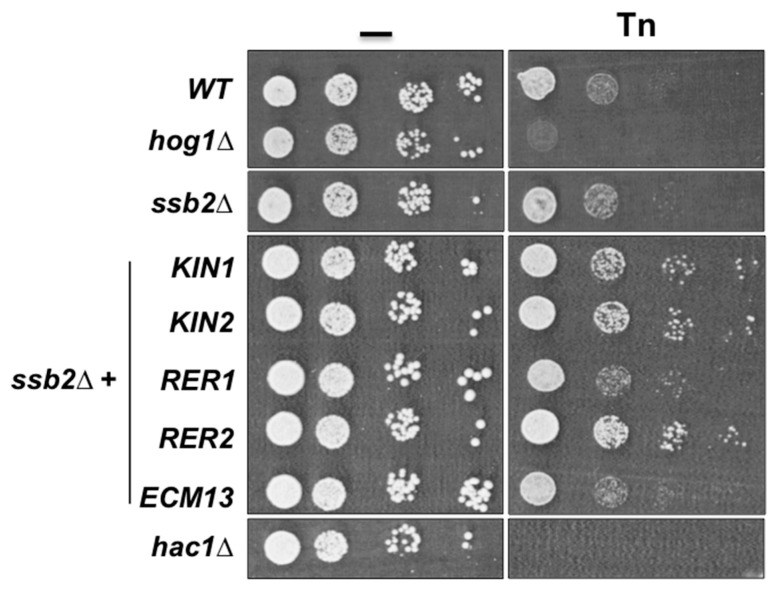
Effect of the inactivation of *SSB2* and overexpression of suppressor genes on Tn sensitivity. The single *ssb2Δ* mutant and transformants carrying the indicated genes were tested in 1.0 µg/mL of Tn as indicated in figure legend 2B. Overexpression was achieved by cloning the indicated genes in the multicopy plasmid YEp352 under the control of their own promoter. Serial dilution assay was performed as indicated in figure legend 2B. Plates were incubated at 30 °C for 72 h and scanned.

Since *SSB2* encodes a chaperon of the Hsp70 family that participates in protein folding and appears to be an important interaction node for Rer2 and Kin1 we evaluated its involvement in the ER stress response. We observed that the mutant lacking Ssb2 showed resistance to Tn at the same level as the wild type strain (Figure 6), however overexpression of *RER2*, *KIN1*, and its paralog *KIN2* in the *ssb2Δ* mutant increased its resistance to Tn, while overexpression of *RER1* and *ECM13* did not (Figure 6). This observation indicates that *RER2*, *KIN1*, and *KIN2* interact genetically with *SSB2*, and also validates the proposed physical interaction network.

### 3.5. The Cis-Prenyltransferase Srt1 also Suppresses the Tn Sensitivity of the Hog1Δ Mutant

**Figure 7:**
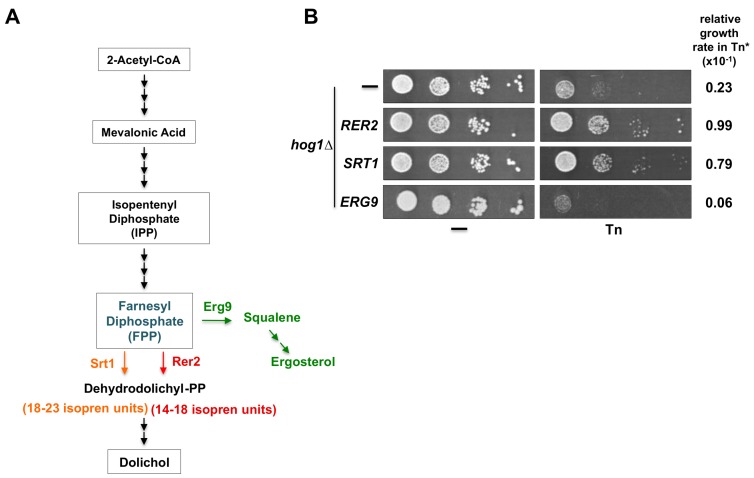
Effects of overexpression of proteins involved in the dolichol and ergosterol synthesis pathways on *hog1Δ* Tn sensitivity. (**A**) Schematic representation of the dolichol and ergosterol synthesis pathways. Farnesyl diphosphate (FPP) is synthesized from 2-Acetyl-CoA through the mevalonic acid pathway. FPP is a shared substrate of Rer2 (and Srt1) and Erg9 to synthesize either dolichol or ergosterol respectively. (**B**) Serial dilution assay of the *hog1∆* strain transformed with the pYES2 vector or with pYES2 carrying *RER2*, *SRT1*, or *ERG9* genes. A spot dilution assay was performed as described above (Figure legend 2B) except that the cells were grown in SRaf medium and then spotted onto SGal or SGal containing 0.5 µg/mL Tn. Plates were incubated at 30 °C for 72 h and scanned. (*) The growth rate in Tn was measured as described in Figure legend 2C.

Tn is a protein glycosylation inhibitor that blocks the activity of the UDP-*N*-acetylglucosamine phosphate transferase (Alg7). This enzyme catalyzes the addition of *N*-acetylglucosamine to dolichol-phosphate, located in the ER membrane. Production of dolichol from farnesyl diphosphate is dependent on the activity of the *cis*-prenyltransferase Rer2 which synthesizes dolichols of 14–18 isoprene units. Yeast cells express a second *cis*-prenyltransferase known as Srt1 (Figure 7A), located mainly in lipid droplets, which synthesizes dolichols of 18–23 isoprene units [41,48]. We tested whether overexpression of *SRT1* would suppress the Tn sensitivity displayed by the *hog1Δ* mutant. We found that *hog1Δ* cells overexpressing *SRT1* grow as well as those expressing *RER2* in a medium containing 0.75 µg/mL Tn (Figure 7B). Accordingly, overexpression of *SRT1* was able to revert the sensitivity of a *pbs2Δ* mutant but not that of the *hac1Δ* mutant, indicating that the suppression process is specific to the HOG1 pathway (not shown). These results suggest that an increase in the cellular concentration of dolichol may revert the Tn sensitivity displayed by the *hog1Δ* mutant.

### 3.6. Hog1-Tn Sensitivity Is Enhanced by Overexpression of ERG9

Farnesyl diphosphate (FPP) is not only a precursor of dolichol but also of ergosterol through a series of reactions initiated by the farnesyl-diphosphate farnesyl transferase encoded by the essential *ERG9* gene (Figure 7A). We set out to determine whether the over-utilization of farnesyl diphosphate for the production of ergosterol would affect growth of the *hog1Δ* mutant in Tn. We found that the sensitivity to 0.5 µg/mL Tn of the *hog1Δ* mutant was enhanced when *ERG9* was overexpressed (Figure 7B). Taking this result together with the observation of the effect of *RER2* and *SRT1* overexpression indicated that the effect of Tn on the *hog1Δ* mutant may in part be dependent on the cellular concentration of dolichol.

### 3.7. The *hog1Δ* Strain Has Reduced Polyprenols Concentration

**Figure 8:**
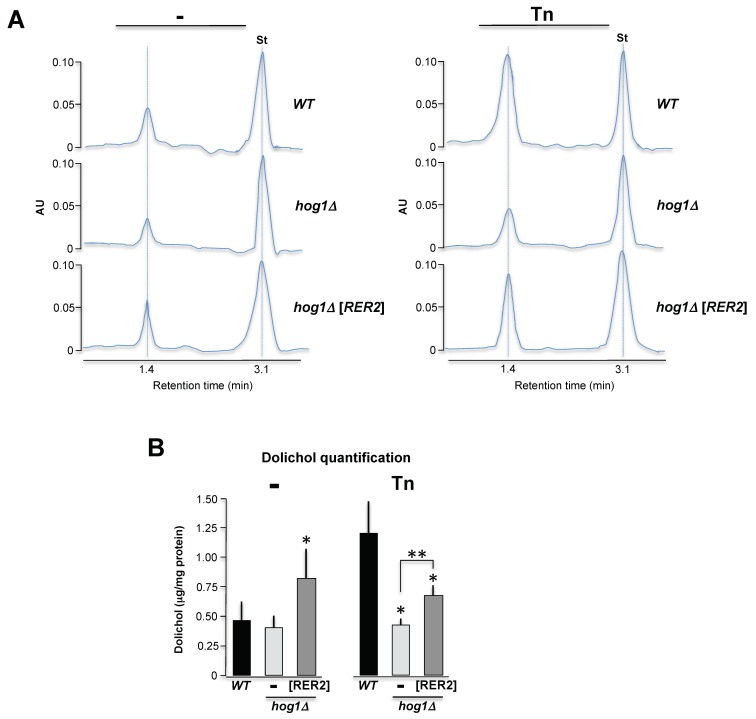
Effect of Hog1 inactivation and Tn treatment on the polyprenols concentration. (**A**) HPLC analysis of dolichols isolated from the wild type, *hog1∆* and *hog1∆*[*RER2*] strains, with or without 1 μg/mL Tn. Dolichol detection was performed by reverse phase HPLC analysis. One representative profile out of three is depicted. 1 μg/μL of standard dolichol (18 isoprene units) was included as internal control (St). (**B**) Dolichol concentration was calculated by determining the area under the curve of the dolichol peak relative to the area under the curve of the standard peak (St) and adjusted by protein concentration (See supplemental Table S4). Mean values (± SD) were calculated from three independent experiments. One asterisk indicates statistical significance regarding the WT strain. Two asterisks indicate statistical significance between *hog1∆* and *hog1∆*[*RER2*] strains.

Based on the previous observations, we hypothesized that the deficient growth of the *hog1Δ* mutant in media containing Tn could be due to an impairment in the dolichol synthesis. We determined the polyprenol content by liquid chromatography (HPLC) in wild type, *hog1Δ*, and the *hog1Δ* overexpressing *RER2* strains treated or not treated with Tn. It was possible to detect in the three strains an enriched polyprenol that may range from 12 to 15 isopren units (Figure 8A). In the wild type strain, the Tn treatment induced approximately a two-fold increase in dolichol concentration (Figure 8B). The same treatment however was unable to induce an increment of dolichol in the *hog1Δ* strain. Overexpression of *RER2* in the *hog1Δ* mutant produced an increment of dolichol, which was statistically significant in both treatments (Figure 8B). Taking together, these results suggest that the *hog1Δ* strain has a strong defect in the polyprenol metabolism under stressful conditions and that this defect is partially compensated with the *RER2* overexpression. 

## 4. Discussion

The isolated suppressors of Hog1 Tn sensitivity allowed us to identify genes whose products participate in a restricted number of cellular processes. According to the SGD and GO geneontology databases, these include protein glycosylation (Rer2 and Alg7); vesicle transport and exocytosis (Rer1, Rer2, Kin1, and Kin2); cell wall organization or biogenesis (Gfa1, Nab6, and Ecm13); and cell detoxification processes (Yor1). The limited number of specific suppressors that were detected indicates that the mechanisms by which Hog1 participates in a protective response to ER stress may be limited to very few pathways. It is interesting that with this screening we did not detect proven physical interactors of Hog1. This can be explained by the fact that most interaction assays with Hog1 are carried out under hyperosmotic conditions. This observation suggests that the suppressor proteins may not be phosphorylation targets of Hog1 and that Hog1 may play a regulatory role in the Tn response through one or more intermediates with the suppressor proteins. Several of these intermediates participate in cellular processes related to stress conditions. However, it has been demonstrated that the kinase activity of Hog1 is essential to trigger a protective response to ER stress inducers [13,49]. Accordingly, we found putative Hog1 phosphorylation motifs (S/T-P) [50,51] in some suppressor proteins except Rer1, Rer2, and Alg7. Kin1 and Kin2 have 3 and 5 putative S/T-P phosphorylation sites respectively, while Nab6, Yor1 and Gfa1 contain one site each [52] (https://phosphogrid.org/). Although we cannot rule out that Hog1 may phosphorylate some suppressor proteins our interaction assay disregard this possibility, however a more extensive interaction study would be suitable.

It is interesting that none of the suppressors found plays a role in gene transcription and that all of them have extra-nuclear activities, which is in agreement with the observation that Hog1 is not transported into the nucleus in response to Tn stress [13,49]. Additionally, the specific suppressors found in this study were able to revert the Tn sensitivity shown by the *pbs2Δ* and the *ssk1Δ* mutants. This indicates that these suppressors may participate in one or several pathways made up of extra-nuclear proteins, including components of the phosphorelay pathway. Accordingly, a version of Hog1 that has been anchored to the plasma membrane by means of a CAAX motif was able to provide full ER stress protection [49]. All these observations support a model in which Hog1 shows regulatory cytoplasmic activity in order to counteract the stress caused by Tn.

The functions that Alg7, Gfa1 and Yor1 have in the cell make them logical candidates to provide cellular protection against Tn. In fact, they revert the Tn sensitivity not only of the *hog1Δ* mutant but also that of the *hac1* mutant. The increased concentration of Alg7, the UDP-*N*-acetylglucosamine phosphate transferase that catalyzes the addition of *N*-acetylglucosamine to dolichyl-phosphate [32], would titer the drug due to the fact that Tn and the enzyme substrate UDP-GlcNAc have a similar structure and compete for the enzyme’s active site. Similarly, the overexpression of Gfa1, an amidotransferase that participates in the formation of glucosamine 6-P, which is a precursor of UDP-GlcNAc [34], would increase the concentration of UDP-GlcNAc, counteracting the inhibitory effect of Tn on Alg7. Finally, the overactivity of the ABC transporter Yor1 would help to eliminate the Tn from the cell as it does with different organic compounds and xenobiotics [36,53]. The finding of these suppressors is an indication that the screening has been performed properly since their function is related to the action mode of Tn.

Within the specific suppressors, *KIN1* and *KIN2* encode serine/threonine kinases. They are part of the Snf1 kinase family of the Ca^2+^/calmodulin-dependent kinase II (CaMK), involved in the regulation of cell polarity and exocytosis, as well as in the regulation of the septin cytoskeleton and cell wall [39,40]. Their *hog1Δ*-suppression activity may be related to one of those functions; however, it has been recently assigned a new role for the Kin kinases. It appears that Kin1 and Kin2 may have a role in the Ire1-mediated targeting and processing of *HAC1* mRNA, thus positively regulating the UPR [54]. Interestingly, as detected by Anshu et al. [54] and in our assays, Kin1 and Kin2 are not totally redundant since a lack of Kin2 produced high sensitivity to Tn; in contrast, a lack of Kin1 barely affected the cellular response to Tn. Since the *HAC1* processing appears to be normal in a *hog1Δ* mutant [13], it is interesting to have found Kin1 and Kin2 as specific suppressors of Hog1. Our epistatic experiments suggest that Hog1 and at least Kin2 act in parallel pathways regarding ER stress. Taken together, these observations suggest that the suppression activity of Kin2 (and perhaps that of Kin1) is more closely related to its function in cell wall regulation and exocytosis, than to its role in regulating the UPR [40,54].

Rer1 and Rer2 were also found as specific Hog1-suppressors. Unlike Kin1 and Kin2, these proteins do not contain putative Hog1 phosphorylation motifs. Rer1 is localized in the early region of the Golgi apparatus and is involved in the retrieval of proteins from the Golgi and is also essential for the proper localization of proteins in the ER [42,43]. Its suppressor activity could be related to its participation in the ER localization of type II membrane proteins, including Mns1, which is the alpha-1,2-mannosidase that catalyzes the last step in glycoprotein maturation in the ER and is required for the ER-associated protein degradation [55,56,57]. In our assays, disruption of *RER1* moderately impaired the cell in its response to Tn and apparently it is not additive to *HOG1*. The functional relationship between the two proteins in the Tn response requires a more detailed study.

*RER2* encodes the *cis*-prenyltransferase enzyme, which catalyzes the conversion of farnesyl diphosphate (FPP) to dehydrodolichyl diphosphate, the precursor of dolichol, which is the ER lipid where the oligosaccharide required for the *N*-glycosylation is assembled [41]. The suppression activity of Rer2 could be related mainly to its role in increasing dolichol concentration. This observation is supported by the fact that the *cis*-prenyltransferase Srt1, which catalyzes the same reaction, also suppresses the Tn sensitivity of a *hog1Δ* mutant. Rer2 is localized in dots associated with the ER and in lipid particles, while Srt1 appears to be localized only in lipid particles [48,58]. Nevertheless, Rer2 is expressed in early logarithmic phase and Srt1 is expressed in stationary phase, and the products of Srt1 are apparently not converted totally to dolichol and dolichyl phosphate; Srt1 behaves as a multicopy suppressor of *rer2Δ* mutant [48] indicating that in the absence of Rer2, Srt1 may adequately supplement the dolichol pool. The *rer2Δ* mutant has a severe growth defect while the *srt1Δ* mutant grows normally [41]. All of these characteristics imply that Rer2 and Srt1 are not redundant, and that they play different roles in the cell physiology; interestingly, they suppress the Tn sensitivity of the *hog1Δ* mutant almost to the same level. Here we found evidence that in the presence of Tn the dolichol content in a wild type strain increases significantly. This suggests that one mechanism to partially counteract the Tn effect is by overproducing dolichol. This protective mechanism is defective in the absence of Hog1 and is partially compensated by the overexpression of *RER2*. For the *hog1Δ* mutant the increased production of dolichol by *RER2* overexpression will partially suppress the Tn sensitivity. This finding is interesting since it has been observed that a *Trypanosoma brucei* mutant with defects in the *N*-glycosylation pathway accumulates larger amounts of polyprenols compared to its parental strain [59]. The involvement of Hog1 in lipid metabolism has been observed previously, for example, inhibition of sphingolipids and ergosterol biosynthesis activates the Hog1 pathway [60] and stress-mediated activation of Hog1 represses the synthesis of ergosterol [61]. An alternative approach to test that the suppression activity of Rer2 (and Srt1) is related to its function in the synthesis of dolichol was by overexpressing, in the *hog1Δ* mutant, the *ERG*9 gene, which encodes the farnesyl-diphosphate farnesyl transferase [62]. FPP is precursor of squalene, which is eventually converted to ergosterol [63,64]. Indeed, here we found that Erg9 may act as a diverter, converting FPP into squalene and enhancing the Tn sensitivity of the *hog1Δ* mutant. All these evidences indicate that the Hog1 role in the ER stress response could be related in part to its involvement in lipid metabolism including the polyprenol pathway. 

The Nab6 and Ecm13 suppression mechanism is at present difficult to deduce since their function remains undefined. Nab6 appears to bind RNAs that encode proteins for the cell wall [38]. It is present in stress granules and co-purifies with Cap-binding proteins and other translation factors [38,65]. It could be that Nab6 regulates translation of this sort of mRNA in response to stress conditions since we detected that the *nab6Δ* mutant is moderately sensitive to Tn. *ECM13* encodes a protein of unknown function that is upregulated by cell wall damage and it may be involved in cell wall biosynthesis and architecture [44,45]; however the null mutant is as resistant to Tn as the wild type strain.

In summary, the screening of high dosage gene suppressors allowed us to conclude that Hog1 may be performing pleiotropic functions in order to regulate the cellular response to ER stress inducers. To counteract the effects of tunicamycin, the cell requires a number of cellular proteins to act in concert with Hog1. It is of the highest interest to study in detail the functional relationship between the suppressor proteins and Hog1. This study opens the field to the investigation of new functions of this MAP kinase in yeast.

## Figures and Tables

**Table 1:** General and specific suppressors and the cellular process where they participate.

*Gene*	Protein Name	Biological Process	Cellular Component
*ALG7*	UDP-*N*-acetyl-glycosamine-1-P-transferase	Protein N-linked glycosylation	Endoplasmic reticulum
*GFA1*	Glutamine-fructose-6-phosphate amidotransferase	Cell wall biosynthesis	Unknown
*YOR1*	Plasma membrane ATP-binding cassette (ABC) transporter	Xenobiotic transport	Plasma membrane
*NAB6*	Putative RNA binding protein	Binds to poliA RNAs	Cytoplasm
*KIN2*	Serine/threonine protein kinase	Exocytosis	Plasma membrane
*KIN1*	Serine/threonine protein kinase	Exocytosis	Plasma membrane
*RER2*	Cis-prenyltranferase	ER to Golgi vesicle-mediated transport. Protein glycosilation	Endoplasmic reticulum
*RER1*	Retention in the endoplasmic reticulum	ER to Golgi vesicle-mediated transport. Protein retention in the ER lumen. Retrograde vesiclemediated transport, Golgi to ER	COPI-coated vesicle vacuole
*ECM13*	Protein induced by traetment with methoxypsoralen and UVA irradation	Cell wall biosynthesis?	Unknown

## Supplementary Materials

The following are available online at https://www.mdpi.com/2073-4409/8/7/710/s1, Table S1: Yeast strains used in this work, Table S2: List of oligonucleotides used in this work; Table S3: Relative interaction of Hog1 with suppressor proteins, Table S4: Dolichol concentration determined from HPLC profiles.
