# Supplementary material for: Tunicamycin Sensitivity-Suppression by High Gene Dosage Reveals New Functions of the Yeast Hog1 MAP Kinase
Source: Cells. 2019 Jul 12;8(7):710. doi: 10.3390/cells8070710 (PMC6678945; doi:10.3390/cells8070710)
Supplement: Supplementary file 1 [file cells-08-00710-s001.zip › Supp_Files/Supp_Table_1.pdf]

Supplemental Table 1. Yeast strains used in this work.

**Strain**	**Genotype**	**Source**
BY4742 WT	MATα *his3Δ1 leu2Δ0 lys2Δ0 ura3Δ0*	EUROSCARF
hog1Δ	BY4742 *hog1:KanMX4*	EUROSCARF
pbs2Δ	BY4742 *pbs2:KanMX4*	EUROSCARF
hac1Δ	BY4742 *hac1:KanMX4*	EUROSCARF
yor1Δ	BY4742 *yor1:KanMX4*	EUROSCARF
nab6Δ	BY4742 *nab6:KanMX4*	EUROSCARF
kin2Δ	BY4742 *kin2:KanMX4*	EUROSCARF
kin1Δ	BY4742 *kin1:KanMX4*	EUROSCARF
rer1Δ	BY4742 *rer1:KanMX4*	EUROSCARF
ecm13Δ	BY4742 *ecm13:KanMX4*	EUROSCARF
ssb2Δ	BY4742 *ssb2:KanMX4*	EUROSCARF
yor1Δ hog1Δ	BY4742 *yor1:KanMX4 hog1:natMX4*	This study
nab6Δ hog1Δ	BY4742 *nab6:KanMX4 hog1:natMX4*	This study
kin2Δ hog1Δ	BY4742 *kin2:KanMX4 hog1:natMX4*	This study
kin1Δ hog1Δ	BY4742 *kin1:KanMX4 hog1:natMX4*	This study
rer1Δ hog1Δ	BY4742 *rer1:KanMX4 hog1:natMX4*	This study
ecm13Δ hog1Δ	BY4742 *ecm13:KanMX4 hog1:natMX4*	This study
VN Ssb2	BY4741 *SSB2*-VN *URA3*	Bioneer
VN Gis2	BY4741 *GIS2*-VN *URA3*	Bioneer
VN Yor1	BY4741 *YOR1*-VN *URA3*	Bioneer
VN Kin1	BY4741 *KIN1*-VN *URA3*	Bioneer
VN Kin2	BY4741 *KIN2*-VN *URA3*	Bioneer
VN Rer1	BY4741 *RER1*-VN *URA3*	Bioneer
VN Rer2	BY4741 *RER2*-VN *URA3*	Bioneer
VN Ecm13	BY4741 *ECM13*-VN *URA3*	Bioneer
VN Nab6	BY4741 *NAB6*-VN *URA3*	Bioneer
VC Hog1	BY4742 *HOG1*-VC *KanMX4*	This study

Strains are either isogenic to BY4742 or to BY4741. Double mutants and the *HOG1-VC* fused gene were constructed as described in Methods section.
