# Supplementary material for: Tunicamycin Sensitivity-Suppression by High Gene Dosage Reveals New Functions of the Yeast Hog1 MAP Kinase
Source: Cells. 2019 Jul 12;8(7):710. doi: 10.3390/cells8070710 (PMC6678945; doi:10.3390/cells8070710)
Supplement: Supplementary file 1 [file cells-08-00710-s001.zip › Supp_Files/Supp_Table_2.pdf]

Supplemental Table 2 List of oligonucleotides used in this work.

Name	Sequence (5´- 3´)	Anchor site
ALG7F	GGATCCACGCCATAATTCAAC	- 1000
ALG7R	AAGCTTGAGAGGGAATACAAA	+ 1543
GFA1F	GAACACCAATGTGGG	- 530
GFA1R	AGCTTGTAGTGCGCA	+ 2255
YOR1F	GCATGCCGCCTTCTTTAGTTG	- 572
YOR1R	CCCGGGAATGAAAAAGGACCG	+ 4643
NAB6F	GGATCCGGTGGAATGCTCACT	- 947
NAB6R	TCTAGACCGCCTTAGGCTTCC	+ 3467
KIN2F	TGATTCACCGCTTTG	- 467
KIN2R	AGCAGATCTCAGCTT	+ 3537
KIN1F	GAAGGGTCCGGTTAG	- 795
KIN1R	GTGTCTGCTTAGGTCC	+ 3322
RER2F	TCGACAAGATTCG	- 1000
RER2R	GGTTAGTTCCTGC	+ 2840
RER1F	GGATCCCAGAGATGAATA	- 499
RER1R	AAGCTTCTCCATTTATAAATG	+ 753
ECM13F	GCAGTAACGCACGGC	- 548
ECM13R	TCTAGAAAGTGGTGATGCACT	+ 876
DHOG1F	AAAGGGAAAACAGGGAAAACTACAACTATCGTATATAATAAGCTTGCCTTGTCCCCGCCGG	- 40
DHOG1R	GAAGTAAGAATGAGTGGTTAGGGACATTAAAAAAACACGTTTCGACACTGGATGGCGGCGTTAG	+ 1309
HOG1VCF	CGGTAACCAGGCCATACAGTACGCTAATGAGTTCCAACAGGGTCGACGGATCCCCGGGTT	+1266
HOG1VCR	GAAGTAAGAATGAGTGGTTAGGGACATTAAAAAAACACGTTCGATGAATTCGAGCTCGTT	+1309

Anchor site refers to the 5′-nucleotide position of each oligonucleotide
