# Supplementary material for: Tunicamycin Sensitivity-Suppression by High Gene Dosage Reveals New Functions of the Yeast Hog1 MAP Kinase
Source: Cells. 2019 Jul 12;8(7):710. doi: 10.3390/cells8070710 (PMC6678945; doi:10.3390/cells8070710)
Supplement: Supplementary file 1 [file cells-08-00710-s001.zip › Supp_Files/Supp_Table_3.pdf]

Supplemental Table 3.
Relative interaction of Hog1 with suppressor proteins

Venus moiety		Relative interaction[1]	
VC	VN	- Tn	+Tn[2]
Hog1	-	1.00	1.00
	Ssb2	0.92	1.04
	Gis2	0.95	0.58
	Yor1	1.07	0.64
	Kin1	0.89	0.80
	Kin2	1.10	0.76
	Rer1	0.99	0.54
	Rer2	1.34	0.79
	Ecm13	1.05	0.69
	Nab6	1.01	0.89
[1]Relative interaction: Values were normalized respective the Hog/- control			
[2]Cells were treated with 1µg/mL of Tn during 2 h prior to cytometry detection			
