# Supplementary material for: Tunicamycin Sensitivity-Suppression by High Gene Dosage Reveals New Functions of the Yeast Hog1 MAP Kinase
Source: Cells. 2019 Jul 12;8(7):710. doi: 10.3390/cells8070710 (PMC6678945; doi:10.3390/cells8070710)
Supplement: Supplementary file 1 [file cells-08-00710-s001.zip › Supp_Files/Supp_Table_4.pdf]

Supplemental Table 4.
Dolichol concentration determined from HPLC profiles

	Dolichol concentration	
**strain**	**-**	**Tn**
**WT**	0.49 ± 0.17	1.24 ± 0.29
**hog1Δ**	0.37 ± 0.11	0.38 ± 0.05
**hog1Δ [RER2]**	0.85 ± 0.25	0.68 ± 0.07

Dolichol concentration (μg/mg protein). Mean values ± SD from three independent experiments.
